# Supplementary material for: Pyruvate Dehydrogenase Kinase 1 inhibition mediated oxidative phosphorylation enhancement in cartilage promotes osteoarthritis progression
Source: BMC Musculoskelet Disord. 2023 Jul 20;24:597. doi: 10.1186/s12891-023-06585-6 (PMC10357736; doi:10.1186/s12891-023-06585-6)
Supplement: Supplementary file 1 — Additional file 1. [file 12891_2023_6585_MOESM1_ESM.zip › KEGG.Gsea.1653622432464/KEGG_ALANINE_ASPARTATE_AND_GLUTAMATE_METABOLISM.html]

Details for gene set KEGG\_ALANINE\_ASPARTATE\_AND\_GLUTAMATE\_METABOLISM[GSEA]

|  || Dataset | 55457\_collapsed\_to\_symbols.cls.cls #OA\_versus\_normal.cls.cls #OA\_versus\_normal\_repos |
| Phenotype | cls.cls#OA\_versus\_normal\_repos |
| Upregulated in class | normal |
| GeneSet | KEGG\_ALANINE\_ASPARTATE\_AND\_GLUTAMATE\_METABOLISM |
| Enrichment Score (ES) | -0.28878647 |
| Normalized Enrichment Score (NES) | -0.9574675 |
| Nominal p-value | 0.546595 |
| FDR q-value | 0.7216239 |
| FWER p-Value | 1.0 |
Table: GSEA Results Summary

  

Fig 1: Enrichment plot: KEGG\_ALANINE\_ASPARTATE\_AND\_GLUTAMATE\_METABOLISM      
 Profile of the Running ES Score & Positions of GeneSet Members on the Rank Ordered List

  

| SYMBOL | TITLE | RANK IN GENE LIST | RANK METRIC SCORE | RUNNING ES | CORE ENRICHMENT || 1 | GOT1 | glutamic-oxaloacetic transaminase 1 [Source:HGNC Symbol;Acc:HGNC:4432] | 59 | 0.974 | 0.1003 | Yes |
| 2 | GLUD1 | glutamate dehydrogenase 1 [Source:HGNC Symbol;Acc:HGNC:4335] | 1279 | 0.462 | 0.0554 | Yes |
| 3 | GOT2 | glutamic-oxaloacetic transaminase 2 [Source:HGNC Symbol;Acc:HGNC:4433] | 1308 | 0.457 | 0.1024 | Yes |
| 4 | ASL | argininosuccinate lyase [Source:HGNC Symbol;Acc:HGNC:746] | 1318 | 0.455 | 0.1506 | Yes |
| 5 | GLUD2 | glutamate dehydrogenase 2 [Source:HGNC Symbol;Acc:HGNC:4336] | 1554 | 0.420 | 0.1776 | Yes |
| 6 | ALDH5A1 | aldehyde dehydrogenase 5 family member A1 [Source:HGNC Symbol;Acc:HGNC:408] | 1661 | 0.405 | 0.2129 | Yes |
| 7 | DDO | D-aspartate oxidase [Source:HGNC Symbol;Acc:HGNC:2727] | 1839 | 0.380 | 0.2401 | Yes |
| 8 | CPS1 | carbamoyl-phosphate synthase 1 [Source:HGNC Symbol;Acc:HGNC:2323] | 2178 | 0.341 | 0.2505 | Yes |
| 9 | ASS1 | argininosuccinate synthase 1 [Source:HGNC Symbol;Acc:HGNC:758] | 2466 | 0.307 | 0.2613 | Yes |
| 10 | GAD1 | glutamate decarboxylase 1 [Source:HGNC Symbol;Acc:HGNC:4092] | 2534 | 0.299 | 0.2883 | Yes |
| 11 | ASPA | aspartoacylase [Source:HGNC Symbol;Acc:HGNC:756] | 3610 | 0.192 | 0.2255 | No |
| 12 | ADSL | adenylosuccinate lyase [Source:HGNC Symbol;Acc:HGNC:291] | 5685 | 0.017 | 0.0662 | No |
| 13 | GFPT1 | glutamine--fructose-6-phosphate transaminase 1 [Source:HGNC Symbol;Acc:HGNC:4241] | 5847 | 0.005 | 0.0543 | No |
| 14 | GFPT2 | glutamine-fructose-6-phosphate transaminase 2 [Source:HGNC Symbol;Acc:HGNC:4242] | 5849 | 0.005 | 0.0548 | No |
| 15 | NIT2 | nitrilase family member 2 [Source:HGNC Symbol;Acc:HGNC:29878] | 6200 | -0.021 | 0.0298 | No |
| 16 | CAD | "carbamoyl-phosphate synthetase 2, aspartate transcarbamylase, and dihydroorotase [Source:HGNC Symbol;Acc:HGNC:1424]" | 6359 | -0.032 | 0.0210 | No |
| 17 | ABAT | 4-aminobutyrate aminotransferase [Source:HGNC Symbol;Acc:HGNC:23] | 6804 | -0.063 | -0.0067 | No |
| 18 | AGXT | alanine--glyoxylate and serine--pyruvate aminotransferase [Source:HGNC Symbol;Acc:HGNC:341] | 8115 | -0.164 | -0.0908 | No |
| 19 | GPT | glutamic--pyruvic transaminase [Source:HGNC Symbol;Acc:HGNC:4552] | 8411 | -0.186 | -0.0937 | No |
| 20 | GLS | glutaminase [Source:HGNC Symbol;Acc:HGNC:4331] | 8423 | -0.187 | -0.0744 | No |
| 21 | GAD2 | glutamate decarboxylase 2 [Source:HGNC Symbol;Acc:HGNC:4093] | 9249 | -0.252 | -0.1114 | No |
| 22 | PPAT | phosphoribosyl pyrophosphate amidotransferase [Source:HGNC Symbol;Acc:HGNC:9238] | 11534 | -0.505 | -0.2344 | No |
| 23 | GLS2 | glutaminase 2 [Source:HGNC Symbol;Acc:HGNC:29570] | 11547 | -0.507 | -0.1807 | No |
| 24 | ASNS | asparagine synthetase (glutamine-hydrolyzing) [Source:HGNC Symbol;Acc:HGNC:753] | 11913 | -0.574 | -0.1472 | No |
| 25 | ALDH4A1 | aldehyde dehydrogenase 4 family member A1 [Source:HGNC Symbol;Acc:HGNC:406] | 12080 | -0.614 | -0.0940 | No |
| 26 | ADSS2 | adenylosuccinate synthase 2 [Source:HGNC Symbol;Acc:HGNC:292] | 12376 | -0.701 | -0.0414 | No |
| 27 | GLUL | glutamate-ammonia ligase [Source:HGNC Symbol;Acc:HGNC:4341] | 12527 | -0.762 | 0.0290 | No |
Table: GSEA details [plain text format]

  

Fig 2: KEGG\_ALANINE\_ASPARTATE\_AND\_GLUTAMATE\_METABOLISM      
 Blue-Pink O' Gram in the Space of the Analyzed GeneSet

  

Fig 3: KEGG\_ALANINE\_ASPARTATE\_AND\_GLUTAMATE\_METABOLISM: Random ES distribution      
 Gene set null distribution of ES for **KEGG\_ALANINE\_ASPARTATE\_AND\_GLUTAMATE\_METABOLISM**
